# Tertiary lymphoid structures (TLS) identification and density assessment on H&E-stained digital slides of lung cancer

**DOI:** 10.1371/journal.pone.0256907

**Published:** 2021-09-23

**Authors:** Panagiotis Barmpoutis, Matthew Di Capite, Hamzeh Kayhanian, William Waddingham, Daniel C. Alexander, Marnix Jansen, Francois Ng Kee Kwong

**Affiliations:** 1 Department of Pathology, UCL Cancer Institute, University College London, London, United Kingdom; 2 Centre for Medical Image Computing, University College London, London, United Kingdom; 3 Department of Histopathology, Norfolk and Norwich University Hospital, Norwich, United Kingdom; Medical University of Graz, AUSTRIA

## Abstract

Tertiary lymphoid structures (TLS) are ectopic aggregates of lymphoid cells in inflamed, infected, or tumoral tissues that are easily recognized on an H&E histology slide as discrete entities, distinct from lymphocytes. TLS are associated with improved cancer prognosis but there is no standardised method available to quantify their presence. Previous studies have used immunohistochemistry to determine the presence of specific cells as a marker of the TLS. This has now been proven to be an underestimate of the true number of TLS. Thus, we propose a methodology for the automated identification and quantification of TLS, based on H&E slides. We subsequently determined the mathematical criteria defining a TLS. TLS regions were identified through a deep convolutional neural network and segmentation of lymphocytes was performed through an ellipsoidal model. This methodology had a 92.87% specificity at 95% sensitivity, 88.79% specificity at 98% sensitivity and 84.32% specificity at 99% sensitivity level based on 144 TLS annotated H&E slides implying that the automated approach was able to reproduce the histopathologists’ assessment with great accuracy. We showed that the minimum number of lymphocytes within TLS is 45 and the minimum TLS area is 6,245*μm*^2^. Furthermore, we have shown that the density of the lymphocytes is more than 3 times those outside of the TLS. The mean density and standard deviation of lymphocytes within a TLS area are 0.0128/*μm*^2^ and 0.0026/*μm*^2^ respectively compared to 0.004/*μm*^2^ and 0.001/*μm*^2^ in non-TLS regions. The proposed methodology shows great potential for automated identification and quantification of the TLS density on digital H&E slides.

## Introduction

Understanding the host immune response to cancer is a critical area of investigation. This has resulted in the recent introduction of various immunotherapeutic drugs (targeting checkpoint inhibition) in the treatment of lung, renal and skin cancers. In addition, the host immune response is also partly mediated by the Tertiary Lymphoid Structures (TLS) [1]. The latter are discrete entities of lymphoid cells which are recognised on histological H&E-stained sections, as they share some histological features with lymph nodes [2]. In general, TLS are not present under normal conditions in some organs and have been observed in pathogen infection, autoimmune disorders, allograft rejection, and in several types of cancer [3–5]. However, in contrast to autoimmune disorders high densities of TLS in cancers including breast, colorectal and lung cancer, are usually associated with positive patient prognosis, outcomes and improved immunotherapy response [3,6,7].

The presence and importance of TLS in lung cancer were first reported by Dieu-Nosjean *et al*. in 2016 [6], who used immunohistochemistry, gene expression assays, and flow cytometry on large series of lung tumors. They demonstrated that TLS are the sites for the generation of the local and systemic T- and B-cell responses against tumours. Furthermore, in lung cancer, previous studies have identified three maturation stages of TLS culminating in germinal centre formation with significant relevance to patient survival [8]. The authors have described TLS development along the stages of secondary lymphoid organ formation and shown that the second (primary follicle-like stage) and third (secondary follicle-like maturation stages) are dependent on co-expression of CD21, CD23 and CXCL13, but that the first maturation stage (early stage, E-TLS), is characterized by dense lymphocytic aggregates without CD21 and CD23 expression.

TLS density can be assessed in diagnostic H&E sections and can, thus, be easily introduced in routine pathology to serve as a relevant prognostic parameter [9]. TLS are identifiable on H&E sections by histopathologists as discrete entities with curved and smooth outline and contain tightly packed mature lymphoid cells. However, there is no current consensus agreement on the definition of TLS, even though their presence has been evaluated in previous studies by morphology on H&E slides as early as 1990 [10]. For example, it is uncertain if there is a minimum number of mature lymphoid cells in the TLS. Although the lymphoid cells appear much more densely packed in a TLS compared to lymphocytes within normal or inflamed tissue, the minimum density of lymphocytes defining TLS remains unspecified. In addition, the minimum size of a TLS is not agreed. The assessment of TLS density over a large histological area is also very time consuming and subject to interpretation variation. Previous studies have assessed TLS based on representative areas of tumour rather than doing this on the whole tumour area [9,11]. However, only a limited number of studies are in progress for developing automated methods for TLS detection and analysis. Silina *et al*. describes a quantitative pathology approach for the identification and quantification of different TLS maturation stages using seven-color immunofluorescent staining and segmentation algorithms of Inform software [11]. As such, being able to evaluate TLS density across the overall area of the tumour would be more accurate. Furthermore, identification of TLS from routine H&E histological images would allow easier integration into clinical workflows.

Various techniques and methods, based on either hand-crafted or deep learning features, have been developed for digital pathology image segmentation tasks aiming to label regions of an image according to what is being shown and aid pathologists to make diagnostic and treatment processes more efficient. Hand-crafted developed methodologies, where a set of local or global features are extracted, mostly include thresholding methods [12], region growing methods based on seed points growing [13], exploitation of morphology features [14,15], watershed transformation [16,17], active contour models [18,19], Markov Random Fields [20] and dynamic image segmentation methods [21]. On the other hand, many segmentation methods have used deep-learning techniques aiming to address the problem by extracting knowledge directly from the data. There are numerous deep learning methods that have been developed for medical image segmentation. More specifically, these include autoencoders [22,23], deep convolutional neural networks (CNNs) [24,25], cascaded networks [26] and fully convolutional networks [27]. However, the training of complex deep learning networks requires a large number of images and computational power as well as considerable effort and time for their annotation by experts [28].

To this end, in this study, we first propose an automated approach for the identification and quantification of TLS in H&E histological images by applying a method that combines a DeepLab v3+ network, an active contour model and a lymphocytes segmentation approach. Secondly, we aim to translate the visual recognition of TLS by histopathologists into a universally reproducible set of mathematical values for the standardisation of TLS recognition: area occupied by TLS, the minimum number of lymphocytes present and their density (number/unit area). A heat map of lymphocytes is then built, thus allowing us to define TLS in lung tissue (cancer and normal). Based on the above data, we propose formal mathematical criteria for the definition of TLS.

## Materials and methods

**Fig 1:**
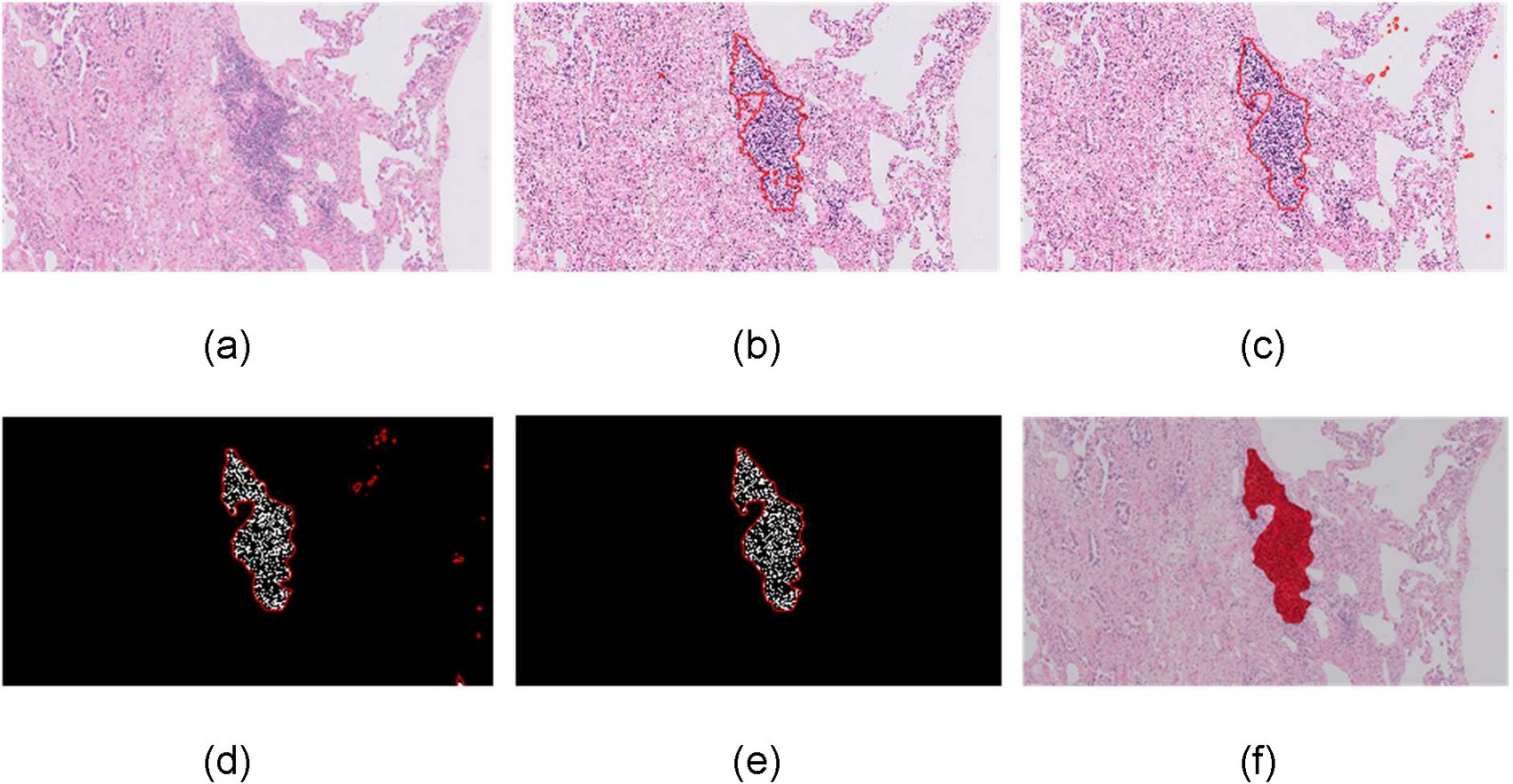
The proposed methodology for TLS detection. a) H&E images feed a DeepLab v3+ network, b) candidate TLS regions are identified, c) an active contour approach is applied for boundary refinement of the TLS regions, d) segmentation of lymphocytes is performed, e) post validation of TLS through rejection of candidate non-TLS regions, f) final detection of TLS regions.

The framework of the proposed methodology for the detection of TLS regions and their lymphocytes is shown in Fig 1. Initially, an H&E image was fed into a modified DeepLab v3+ network for the detection of candidate TLS regions and an active contour model was then applied in order to refine the boundaries of the TLS regions. Then, segmentation of lymphocytes was performed for the identification of the following features: the number of lymphocytes, the size of TLS regions and the number of lymphocytes per unit area of TLS. The estimated features were used for post validation of candidate TLS regions aiming to filter out the false-positive detected TLS regions.

### Identification of the candidate TLS regions

For the identification of the candidate TLS regions, a modified pre-trained DeepLab v3+ model [29] with Inception-ResNet-v2 as the main feature extractor, which employs dropout to avoid overfitting, was utilized. The DeepLab models have been extensively used in the task of semantic medical image segmentation and tested on large volumes of image datasets [30–34]. These models provide a capability in learning multi-scale contextual features through Atrous Spatial Pyramid Pooling (ASPP) and use a decoder module for the refinement of the segmentation results, especially along object boundaries. In this work, the ASPP is a module that employs multiple parallel atrous convolutional layers with different rates to learn multi-scale information of image aiming to identify different sizes of TLS regions and to retain the balance between context assimilation and fine localization. This network was selected due to the good balance it achieves between accuracy and computational complexity. Specifically, Inception-ResNet-v2 outperforms other common configurations with regards to accuracy and complexity [35]. The model was pre-trained on ImageNet and then fine-tuned with training data prepared for this work. Deep neural networks pre-training [36] can be seen as a case of transfer learning [37], in which a neural network further trained on a source dataset is subsequently fine-tuned to a target dataset. The pre-training on the source dataset enables the deep neural networks to learn useful low level features in their first layers, such that good results can be achieved with fewer examples in the fine-tuning stage, which mostly adapts the higher level features in the last layers, hence requiring less labeled training data. It is worth mentioning that color normalization was applied in all the dataset images.

Furthermore, an augmentation method was utilized to enlarge the image samples in order to better fine-tune the DeepLab v3+. Data augmentation artificially enlarges the size of the training dataset by applying spatial warps, which has been proven a very effective strategy in many image analysis tasks [38]. Even though the number of training cases might appear small, we combined the abundant pixel‐level information with pre‐training and with data augmentation in order to increase the variability of training images and to avoid overfitting of the network [39].

Additionally, a modified loss function was defined in order to adjust the model to better deal with the boundaries of TLS. Thus, introducing a weighting factor *w*, we force the model to be sensitive to the TLS boundaries and regions enclosed within the TLS. More specifically, the loss function is defined as follows:

$$Loss=-\sum_{p=1}^{N}w_{p}r_{p}log(t_{p})$$ (1)

where *w*_*p*_, *r*_*p*_ and *t*_*p*_ denote the weighting factors, the reference values and the predicted values at pixel *p* respectively, and *N* is the total number of pixels. Regarding the weighting factors, we set the *w*_*p*_ = 2 when *p* is a TLS pixel and otherwise, we set *w*_*p*_ = 1.

### TLS boundary refinement

Since the lymphoid cells appear in higher density in TLS regions in order to obtain precise TLS contours, we adopted an active contour approach [40] that utilizes local intensity distribution to drive the evolving curve. More specifically, the local intensities within its neighborhood are assumed to follow a Gaussian probability distribution:

$$p_{i,x}(I(y)|m_{i}(x),\sigma_{i}{\left(x\right)}^{2})=\frac{1}{\sqrt{2\pi }\sigma_{i}(x)}exp\left(-\frac{{\left(I(y)-m_{i}(x)\right)}^{2}}{2\sigma_{i}{\left(x\right)}^{2}}\right)$$ (2)

where *m*_*i*_(*x*) and *σ*_*i*_(*x*) are mean and standard deviation of the intensities in each local region. Thus, the local Gaussian distribution fitting energy is estimated as follows:

$$E=-\int_{\Omega }\int_{inside(C)}K_{\sigma }(x-y)logp_{1,x}(I(y)|m_{1}(x),\sigma_{1}{\left(x\right)}^{2})dydx-\int_{\Omega }\int_{outside(C)}K_{\sigma }(x-y)logp_{2,x}(I(y)|m_{2}(x),\sigma_{2}{\left(x\right)}^{2})dydx$$ (3)

where $\Omega \in R^{2}$ represents the image domain, *C* a closed contour and the neighborhood of each pixel is defined by using a truncated Gaussian kernel *K*_*σ*_. Thus, the applied model is able to differentiate between regions with intensity heterogeneity and also between regions with similar intensity means but different intensity variances [41]. The active contour model is initialized by the candidate TLS regions detected in the previous step.

### Lymphocytes segmentation

In digital histopathology the cell segmentation is the task of the automated splitting of microscopic tissue images into segments, which represent individual cells. Many cell segmentation methods have been developed, utilizing both traditional techniques and deep learning methods in the field of medical image analysis. They achieve comparable accuracy rates and they identify single cells through watershed transformation [42], using active contours [43], modelling the cells with a set of circle or ellipses [14,44–46], while many other methods utilize deep neural networks [47–49].

**Fig 2:**
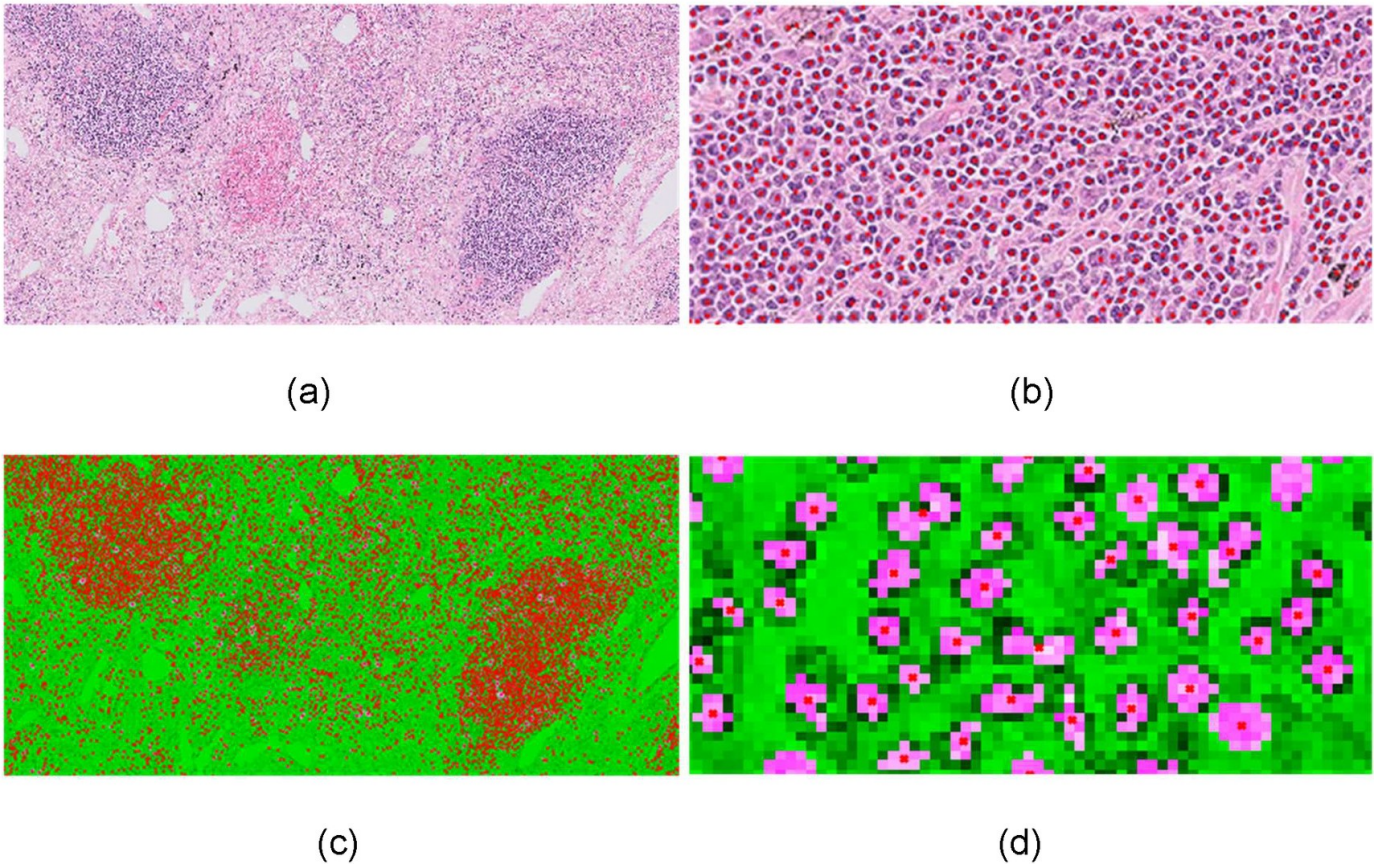
Lymphocyte segmentation. (a) input 10x H&E-stained image, (b) lymphocytes identification -red dots, (c) separation of merged or overlapped cells through the proposed ellipsoidal modelling, (d) detailed image of single-cell segmentation.

In this work, we propose an improved methodology based on an ellipsoidal model [14] that iteratively identifies and counts the cells (Fig 2) aiming to keep good balance between the estimated cells’ shape and overlapping parts of touching cells through a single validation criterion and at the same time to overcome the limitations of previously developed methods [14] that in many cases erroneously reject small touching cells. To this end, initially, input RGB images were converted to grayscale and filtered using a Gaussian filter with a 3×3 kernel in order to remove small artifacts. Furthermore, a histogram equalization filter was applied in order to enhance the differences between lymphocytes and other tissue components. Subsequently, an effective method using an adaptive threshold approach was applied. More specifically, this method set the threshold based on the local mean intensity in the neighbourhood of each pixel. Thus, the formula used for thresholding was defined as follows:

$$O(i,j)=\left\{ \begin{array}{c} 255,I(i,j)\geq T\\ 0,I(i,j)<T \end{array}\right.$$ (4)

where *O*(*i*,*j*) is the resulting pixel of output image at (*i*,*j*), *I*(*i*,*j*) is the pixel of the input image and T is the selected local threshold value. In the binary image, in order to suppress small artifacts, morphological operations consisting of erosion, dilation and removal of small elements were applied.

For the separation of touching cells an improved ellipsoidal modelling approach is proposed. Initially, we estimated the distance transformation of the binary image *M* of *p* pixels that represents the connected cells and we estimated the regional maxima of this. Considering that the number and location of local maxima corresponds to these of nuclei, we rejected the touching maxima. The remaining maxima comprise the list of candidate seeds. Based on the hypothesis that cells can be spatially modeled as ellipsoids *E*_*C*_, the pixels of cells were then modeled using a Gaussian distribution. More specifically, a Gaussian mixture model was applied with the number of clusters *C* being equal to that of candidate seeds and the mixture parameters, namely mean and variance, to be estimated using the expectation-maximization (EM) algorithm. For the initialization of the EM algorithm we used *k*-Nearest Neighbor classification using Euclidean distance as the distance metric in order to estimate the initial parameters. The EM is an iterative method consisting of two steps: (i) expectation, which computes the likelihood with respect to the current estimates and (ii) maximization (Eq 5), which maximizes the expected log likelihood (Eq 6) as follows:

$$Q(\theta |\theta^{\left(t\right)})=E_{Z|X,\theta^{\left(t\right)}}[logL(\theta ;Χ,Ζ)]$$ (5)

$$\theta^{\left(t+1\right)}=arg\;\underset{\theta }{\max}Q(\theta |\theta^{\left(t\right)})$$ (6)

where *Q* is the expected values of the log likelihood function *θ*, *X* is the pixel coordinates, *Z* is the latent variables and *θ*^(*t*)^ is the current parameters.

Having estimated the ellipsoidal models of cells, we need to identify the optimal number of seeds rejecting or approving the candidate seeds from the previously estimated list. Thus, in this approach, we proposed a single fitness validation criterion estimating this for the total number of combinations of candidate seeds, aiming to accurately identify the total number of cells. The criterion takes into account the foreground, the background and the overlapping cell areas that are included by the estimated ellipses and the total area of the extracted ellipses. More specifically, the total area covered by the estimated ellipses is defined as follows:

$$E=\sum_{p\in M}E_{C}(p)$$ (7)

the foreground area of the binary image *M* that is included by the estimated ellipses is defined as follows:

$$A_{F}=\sum_{p=1}M(p)E(p)$$ (8)

the area of the background area of the binary image *M* that is included by the estimated ellipses is defined as follows:

$$A_{B}=\sum_{p=1}\left[1-M(p)\right]E(p)$$ (9)

and the overlapping parts of the ellipses of the touching cells for the total number of the identified ellipses is defined as follows:

$$A_{T}=\sum_{i=1}\sum_{p=1}E_{C_{i}}(p)\cap E_{C_{j}}(p),j=1,j\neq i$$ (10)

Based on the calculation of these metrics, we estimated the fitness degree of the estimated ellipsoidal components against the 2D cell data and we selected the candidate seeds that maximize the following:

$$S=\max\left(\frac{A_{F}-A_{B}-A_{T}}{E}\right)$$ (11)

The final segmentation of the clustered cells was performed by applying Bayesian classification which assigns each pixel *p* to cluster *C*_*i*_ with the maximum posterior probability. Finally, as lymphocytes typically have small (7–10*μm*), round, and dark nuclei with little cytoplasm, which is distinctive from malignant cells or stromal cells [50], we used morphological and textural features, namely size and shape cells, the average value and the skewness of the intensity histogram of the cell, in order to reject the non-lymphocytes.

### Post validation of candidate TLS regions

Following the candidate TLS refinement and lymphocytes detection, the falsely detected candidate TLS regions were rejected, in order to validate the identified candidate TLS regions and decrease the false positive TLS identification rates. More specifically, hypothesizing that the lymphocytes density of TLS regions is much higher than lymphocytes within the rest of a tissue, 3 features were extracted and used for the rejection of candidate non-TLS regions. To this end, the number of lymphocytes, the size and the number of lymphocytes per unit area of each candidate TLS region were extracted. After the estimation of the features, an SVM classifier was deployed towards the aim of arriving at a final decision regarding whether an identified candidate TLS region is an actual TLS region or a false-positive candidate case.

### Dataset description

Formalin-fixed paraffin-embedded tissue, surplus to diagnostic purposes, was obtained from patients undergoing lung cancer resection. Informed consent was obtained from the donor prior to surgery for use of surgically-excised tissues for research purposes. This study was approved by the local Ethical Committee of the University of East Anglia (Ref No. 2017/2018–119 HT). Histological cases were retrieved from the archive of the Norfolk and Norwich University Hospital histopathology department. Tumours were classified according to the 2015 WHO classification [51]. For each patient, TLS assessment was based on a representative tissue block, with adequate tumour material and interface between normal and tumor tissue well represented. Annotation was performed on tumour tissue slides from 18 patients with primary lung cancer. There were 8 tumours, of which there were 14 adenocarcinoma (5 acinar predominant, 1, papillary, 2 solid predominant and 6 lepidic predominant), 3 squamous cell carcinomas and 2 sarcomatoid carcinomas. The age range of the patients was 54–82 years old, with an average of 69 ± 2.4 years. The tumour size was between 8 and 62 mm, with an average of 28 ± 6.4. TLS was defined as all dense lymphocytic aggregates and 144 TLS were annotated by 2 histopathologists (FK, MDC) with 100% concordance. To assess the generalizability of the model, two datasets (D1 and D2) were created consisting of 5 and 13 patients respectively for internal training-validation and further validation in independent populations.

## Results

For the evaluation of the proposed method we conducted extensive tests using the two datasets. Initially, through the first dataset, we internally validated the efficiency for TLS identification of the proposed methodology by performing an ablation analysis and leave-one-out cross-validation and then through the second dataset we externally validated the generalizability of the proposed model in a different population. Furthermore, we compared the efficiency of identification of TLS regions, using state of the art approaches. Finally, for the identified TLS regions for both datasets, we used box plots to show the range of the number and density of lymphocytes as well as the TLS area.

### Identification of TLS regions

The presented TLS identification and density assessment methodology comprised three main components, namely DeepLab model, its active contour approach and post-validation processing. The applied DeepLab v3+ model was used to identify the candidate TLS regions in a semantic image segmentation task, the active contour approach for boundary refinement of the candidate TLS regions, while the post-validation scheme was used to reject the candidate non-TLS regions.

**Fig 3:**
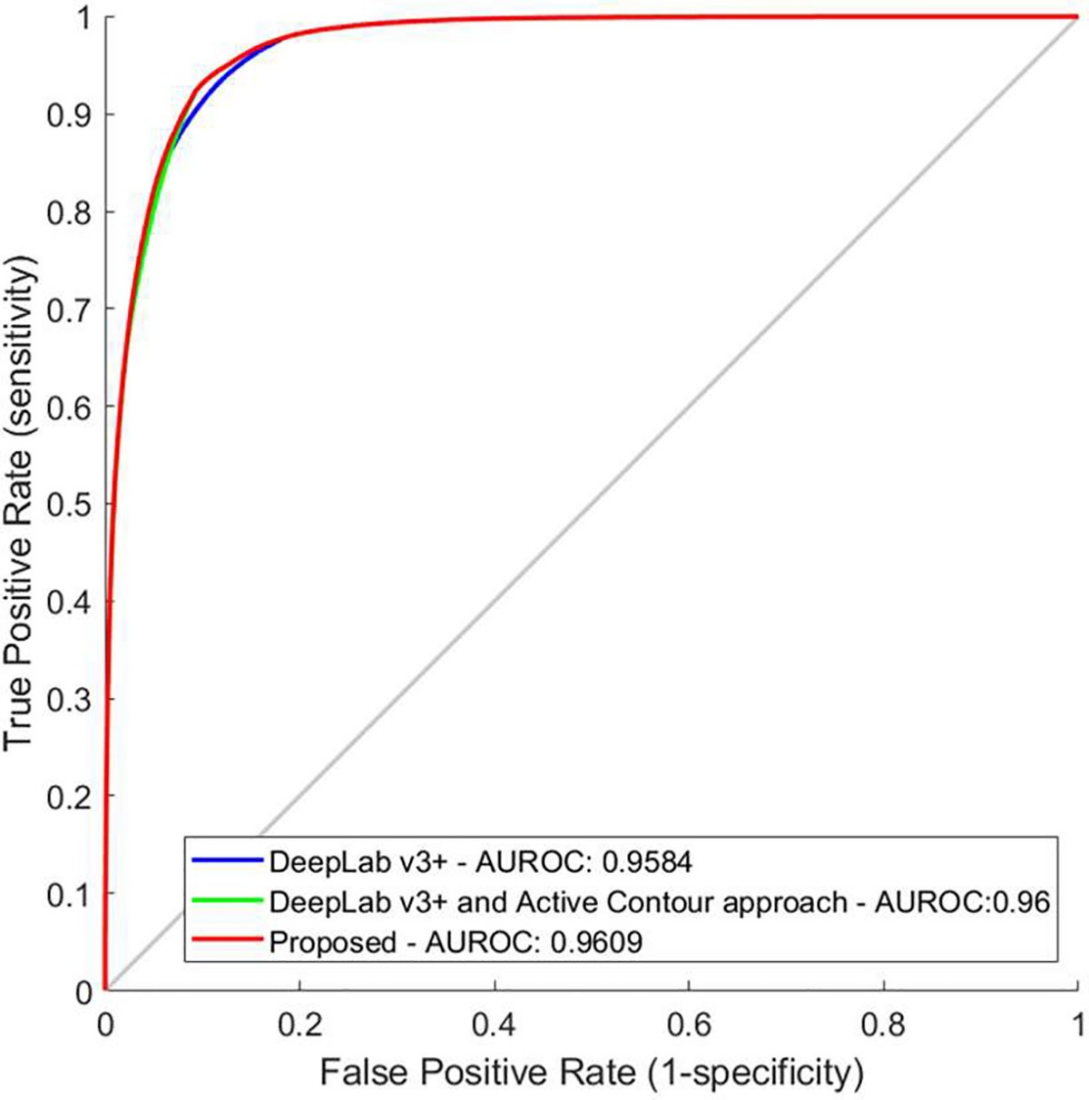
Ablation analysis and performance on tissue slide‐based analysis of the a) DeepLab v3+ model, b) DeepLab v3+ and active contour approach and c) proposed methodology. AUROC, area under the receiver operating characteristic curve as shown in the image; FPR, false positive rate; TPR, true positive rate.

Initially, for the evaluation of the components of the proposed methodology, in the first dataset we performed tissue slide-level leave-one-out analysis. Thus, we found that the DeepLab v3+ model achieves a performance that reaches an Area under the Receiver Operating Characteristic (AUROC) curve of 0.9584. More specifically, defining sensitivity levels, at 95%, 98% and 99%, the DeepLab v3+ model achieves 85.79%, 80.95% and 74.98% specificity rates respectively. In addition to the DeepLab v3+ model, the use of boundary refinement improves the overall performance reaching an AUROC of 0.96. This slight increase in AUROC is translated into higher specificity rates at the predefined sensitivity levels, reaching 86.97% specificity at 95% sensitivity, 80.97% specificity at 98% sensitivity and 74.99% specificity at 99% sensitivity level. The adoption of the post-validation scheme and rejection of the candidate non-TLS regions improves the performance further, reaching an AUROC of 0.9609. Thus, the corresponding specificity and sensitivity rates for the proposed model are 87.02% specificity at 95% sensitivity, 80.97% specificity at 98% sensitivity and 74.99% specificity at 99% sensitivity level (Fig 3).

**Fig 4:**
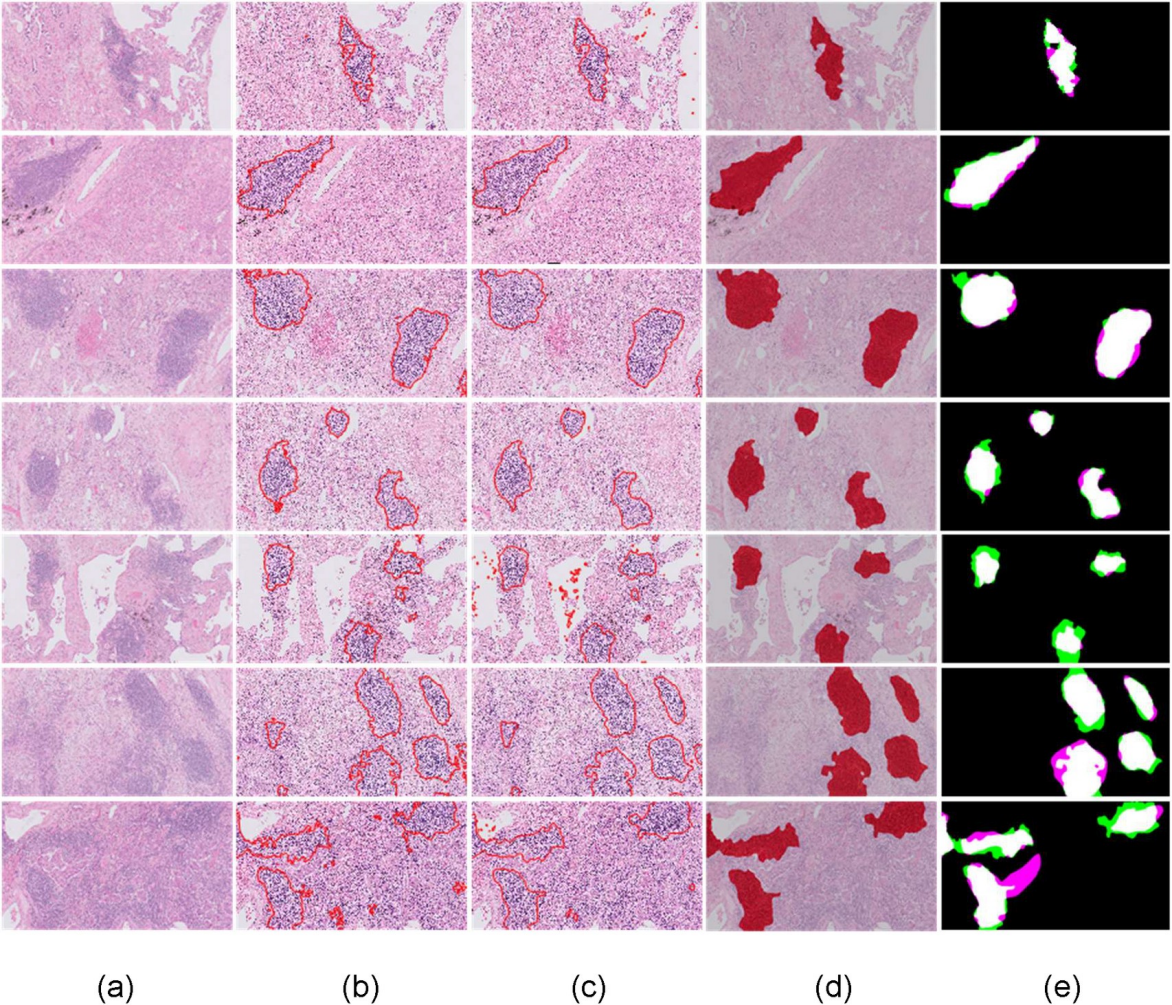
Identification of TLS regions and density of lymphocytes. a) input 10x H&E-stained image, b) DeepLabv3+ identified TLS regions, c) application of active contour model d) post validation of candidate TLS regions—detected TLS regions e) identified TLS regions (classification threshold at 0.5) in comparison with ground truth: White color (True Positive), green color (False Positive), magenta color (False Negative).

Furthermore, in order to confirm that the performance of the proposed methodology remains robust, we carried out an external validation analysis using the second dataset. To this end, we used the first dataset as the training dataset, and the proposed methodology reaches an AUROC equal to 0.9589. More specifically, it achieves 92.87% specificity at 95% sensitivity, 88.79% specificity at 98% sensitivity and 84.32% specificity at 99% sensitivity level. The qualitative results of Fig 4 demonstrate that the total number of the components used in this methodology contribute to the overall accuracy of TLS identification. Based on the input 10x H&E-stained images (Fig 4A), the DeepLab v3+ accurately detects the candidate TLS regions (Fig 4B). Then the active contour approach obtains precise TLS boundaries (Fig 4C) while the post-validation processing step rejects candidate non-TLS regions (Fig 4D) that have partially similar characteristics to TLS regions.

### A comparison of state-of-the-art methods

**Fig 5:**
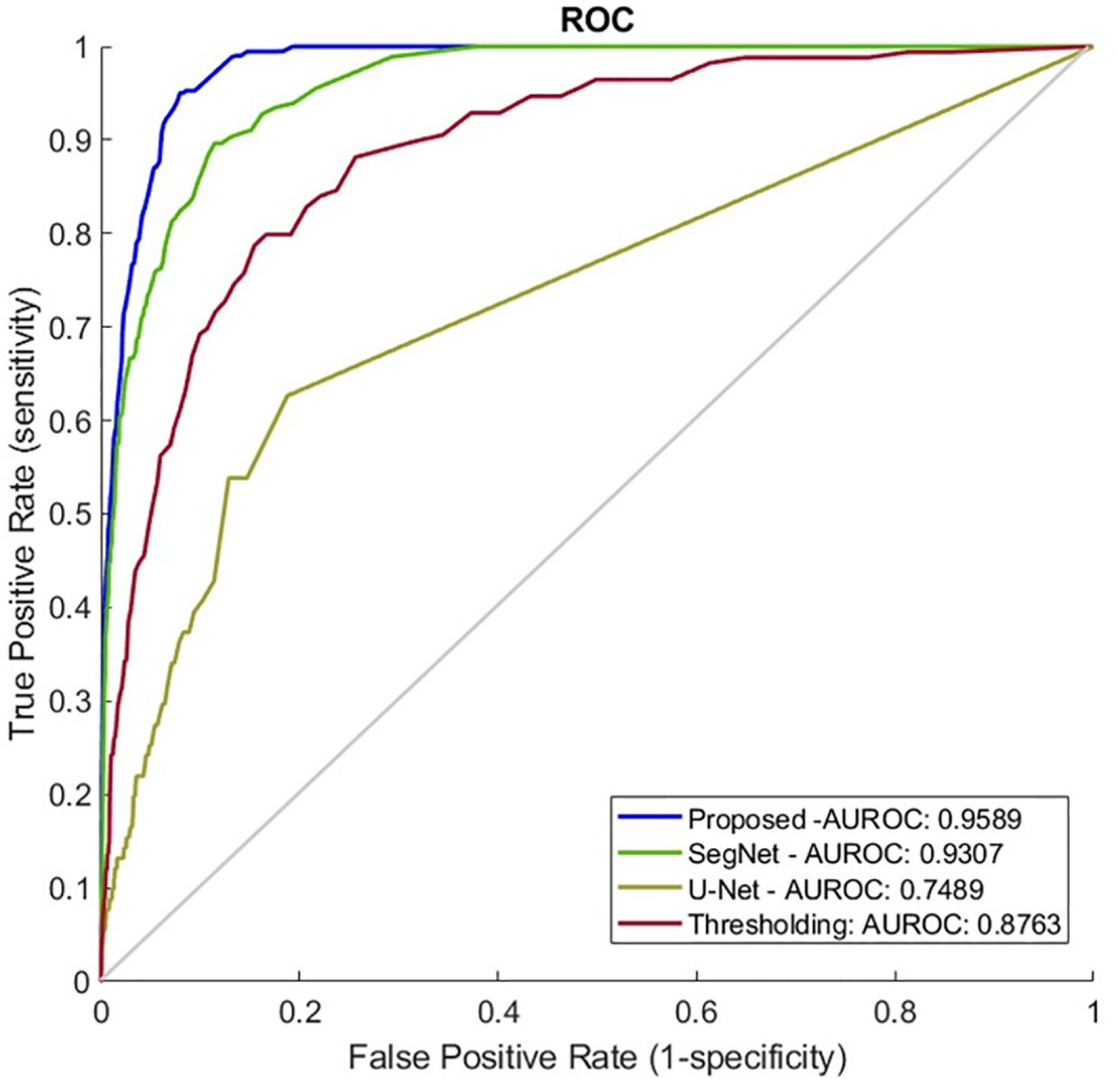
Performance comparison on external validation analysis. AUROC, area under the receiver operating curve as shown in the image; FPR, false positive rate; TPR, true positive rate.

**Fig 6:**
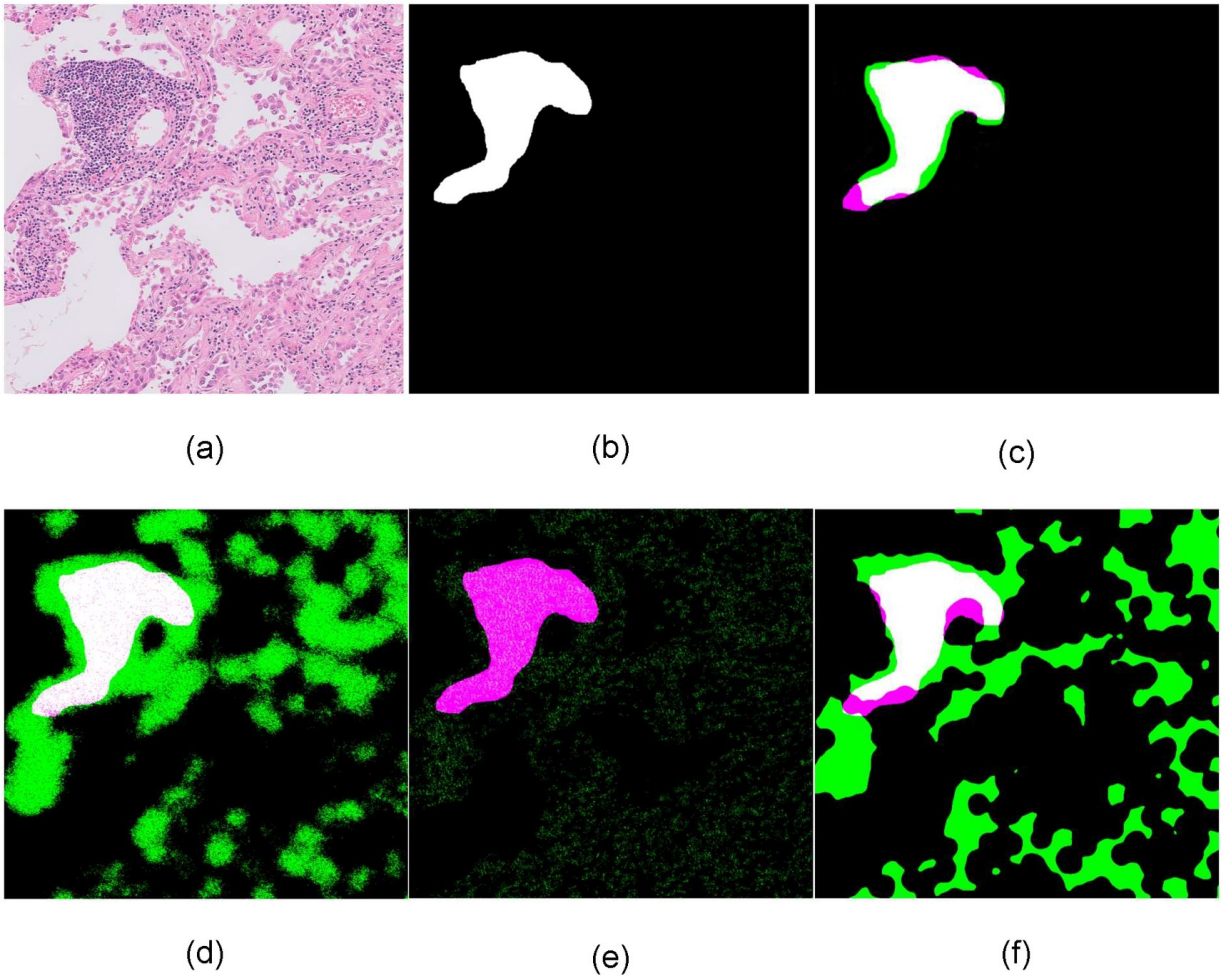
Qualitative comparison on tissue slide‐based analysis. a) input 10x H&E-stained image, b) ground truth c) DeepLabv3+ identified TLS regions, d) SegNet identified TLS regions, e) U-Net identified TLS regions, f) lymphocytes density thresholding identified TLS regions, in comparison with ground truth: White color (True Positive), green color (False Positive), magenta color (False Negative).

For the validation of the proposed methodology, three state of the art methods were deployed. Deploying a SegNet model [52], a U-Net model [53] and a classic lymphocytes-based thresholding method [54] the proposed method outperforms these (Fig 5) identifying more accurate the TLS regions (Fig 6).

More specifically, the SegNet is a CNN architecture for semantic segmentation proposed by researchers at University of Cambridge [52]. The main SegNet applications regarding segmentation tasks such as semantic segmentation of prostate cancer [55], gland segmentation from colon cancer histology images [56] and brain tumor segmentation from multi-modal magnetic resonance images [57]. For the evaluation of the SegNet in the task of TLS identification, we used the same protocol as the proposed methodology and we found that its performance in the external validation set reaches an AUROC of 0.9307. This is translated to 80.87% specificity at 95% sensitivity, 72.14% specificity at 98% sensitivity and 70.12% specificity at 99% sensitivity level.

Then we chose U-Net, a popular deep-learning-based method to solve microscopy image segmentation problems [53]. Deploying the U-Net we found that its performance reaches an AUROC of 0.7489 and 15.2% specificity at 95% sensitivity level. It is worth mentioning that in contrast to the proposed network the U-Net was not pre-trained. This might explain the latter’s insufficient capture of the experimental variation. Finally, we applied a thresholding method [54] aiming to distinguish regions with contrasting density lymphocytes levels. The thresholding method reaches an AUROC of 0.9021 and 57.75% specificity at 95% sensitivity, 38.71% specificity at 98% sensitivity and 22.41% specificity at 99% sensitivity level.

### Criteria for the definition of TLS regions

**Fig 7:**
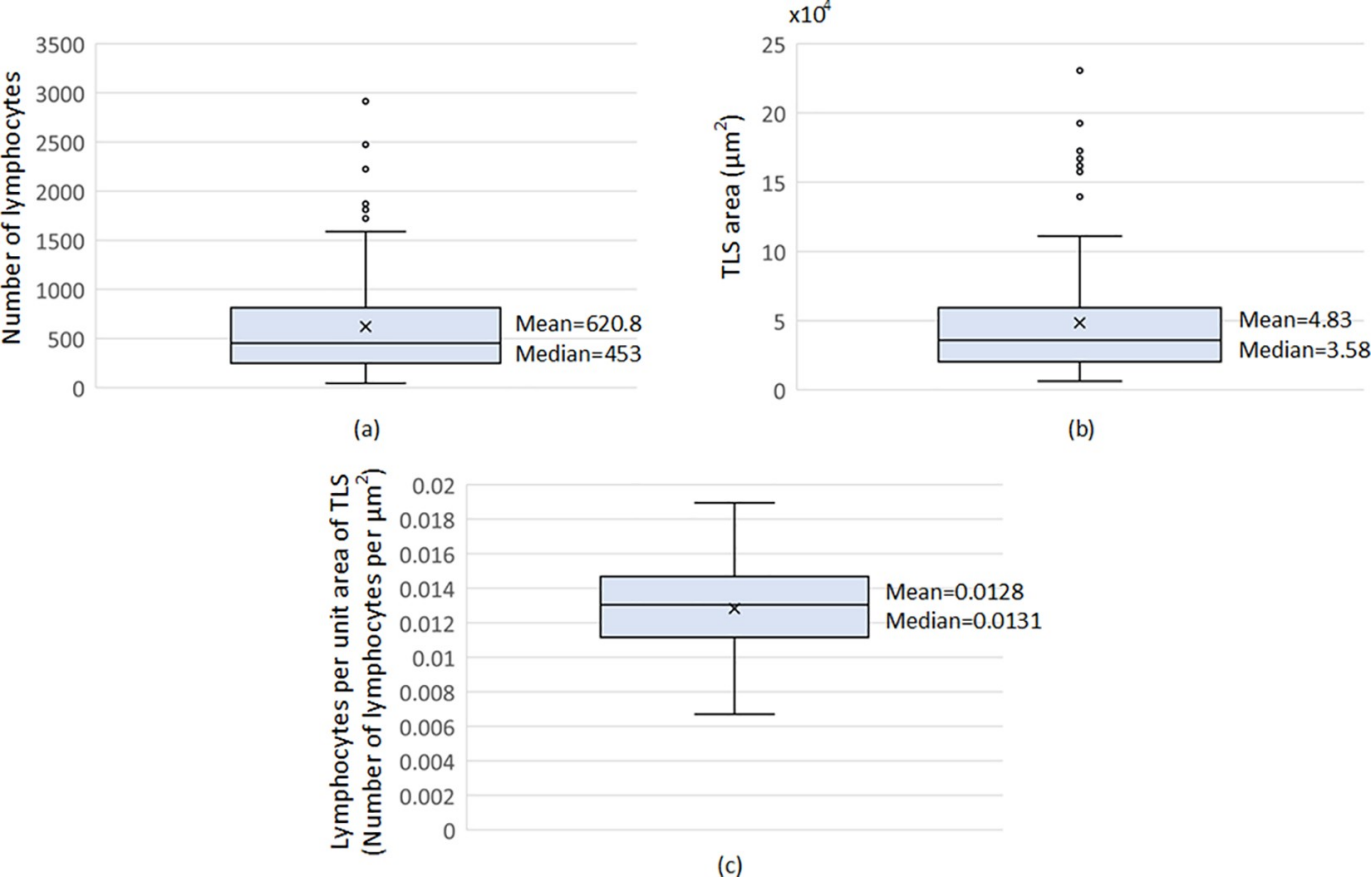
Box plots for (a) the number of lymphocytes, (b) size and (c) density (number of lymphocytes per unit area of the TLS regions) for the hematoxylin and eosin (H&E) stained histological slides included in both of D1 & D2.

In this work, for the standardisation of TLS recognition, we aim to translate the visual recognition of TLS by histopathologists into a universally reproducible set of mathematical values (Fig 7). Thus, through the lymphocytes segmentation procedure, we show that the TLS regions include a minimum number of 45 lymphocytes and that the minimum area of TLS regions is 6,245*μm*^2^. The mean number of lymphocytes in TLS regions is 620.8 while the mean area of TLS regions is 48,387*μm*^2^. The largest number of lymphocytes in TLS regions is 2936 and the largest area of TLS regions is 230,604*μm*^2^. The minimum number of lymphocytes per area of TLS regions is 0.0074/*μm*^2^ with a mean and standard deviation of 0.0128/*μm*^2^ and 0.0026/*μm*^2^ respectively and a maximum of 0.0189/*μm*^2^.

**Fig 8:**
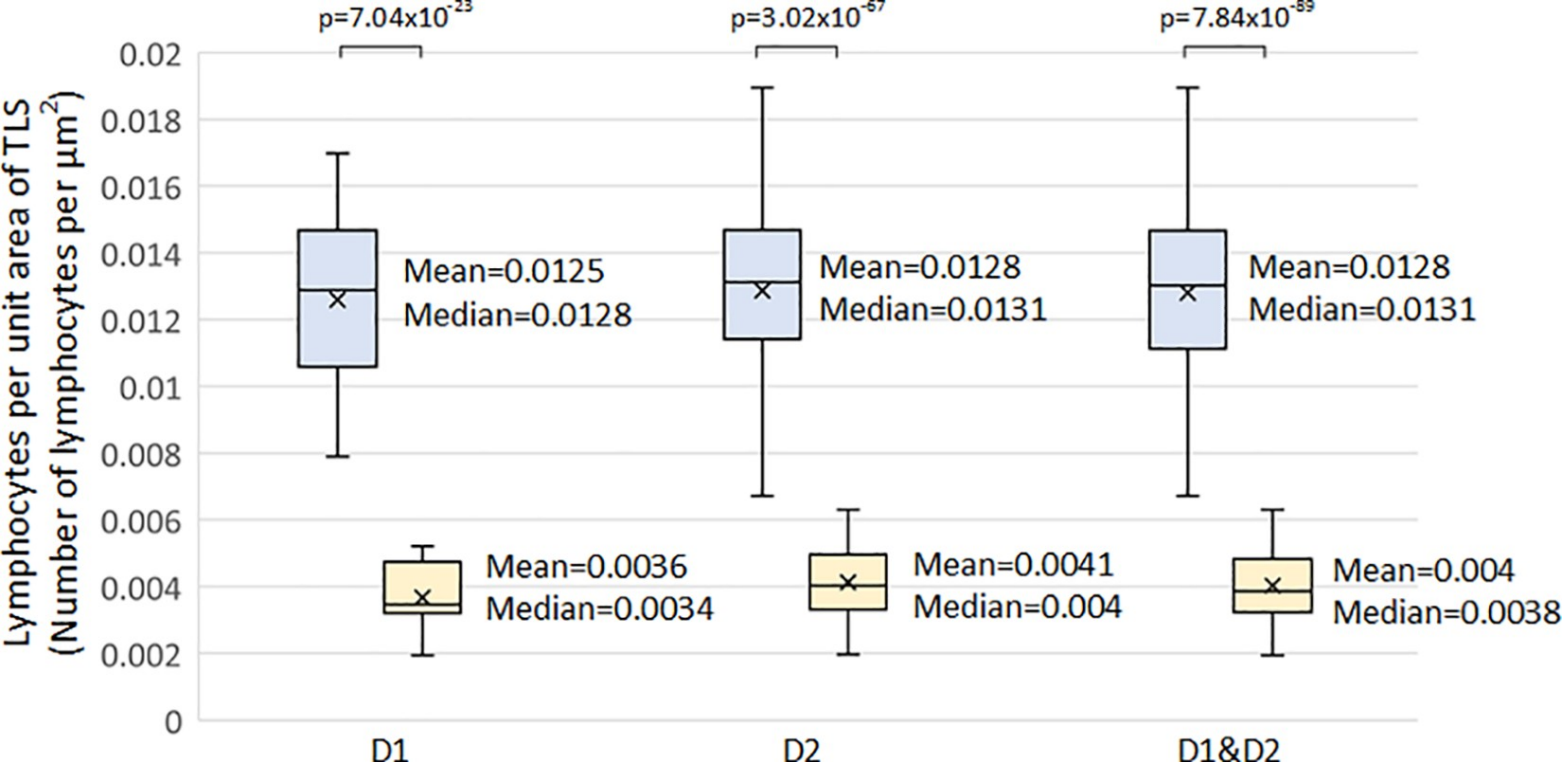
Box plots comparing lymphocyte density between the identified TLS regions and non-TLS regions in hematoxylin and eosin (H&E) stained histological slides included in dataset 1 (D1), in dataset 2 (D2) and in both of them (D1&D2).

**Fig 9:**
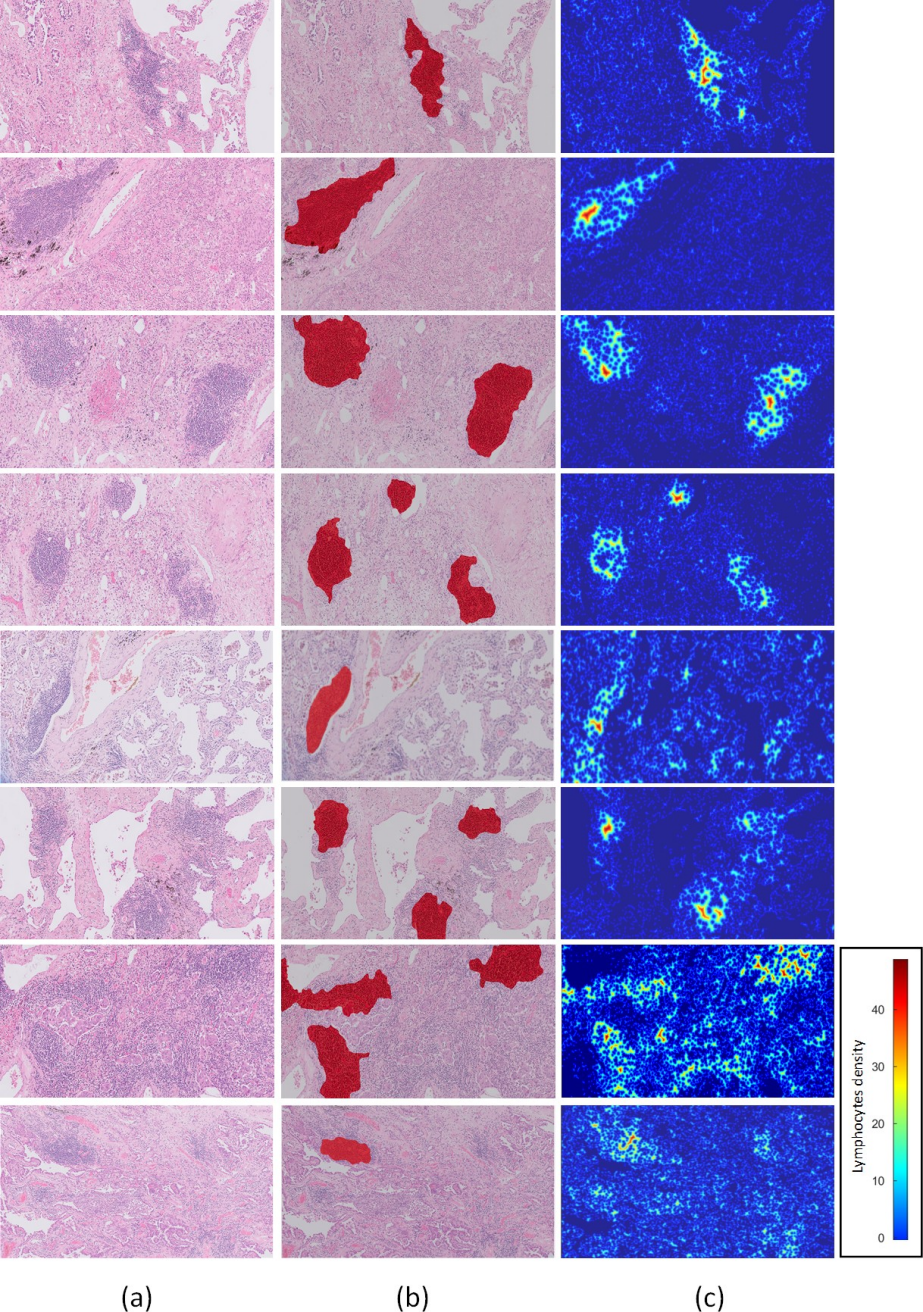
Identification of TLS regions and density of lymphocytes. a) input 10x H&E-stained image, b) detected TLS regions c) heat maps of lymphoyctes.

It is worth mentioning that the aforementioned criteria were defined through the analysis of TLS regions in both datasets created. In additional analyses, we carried out tests for the comparison between the lymphocyte’s density values of identified TLS and that outside of TLS regions. Thus, in the comparison of the lymphocytes density values of identified TLS regions with the values outside of the TLS regions we identified that the minimum number lymphocytes of TLS regions is 0.0019/*μm*^2^, with a mean and standard deviation of 0.0040/*μm*^2^ and 0.0010/*μm*^2^ respectively and a maximum of 0.0063/*μm*^2^ (Fig 8). Finally, it is worth mentioning that for the evaluation of the proposed lymphocytes segmentation method, we manually annotated four hundred cells. Thus, the method was compared with an ellipsoidal model [14] and the classical watershed algorithm outperforming these with a correctly segmented rate of 91.5% in contrast to 89.5% and 86% respectively. Thus, heat maps of lymphocytes were constructed from the input 10x H&E-stained images and their corresponding detected TLS regions (Fig 9). In the heat maps, the dark blue color represents the background and the other cells, while the lighter blue to red colors represent the lymphocytes from lower to higher density. Thus, it is clearly shown that TLS regions were easily recognized within the lung cancer tissue from the lymphocytes heatmap.

## Conclusion

Histologically, the TLS are recognised as mature and tightly packed lymphoid cells forming discrete entities with a smooth circular outline. Previous studies have determined the density of TLS based on the expression of high endothelial venules, DC Lamp expressing cells and other markers. However, based on our diagnostic experience, this is likely to represent an underestimate of the density of TLS. The TLS, detected by immunohistochemistry, represent only a subset of all the TLS which can be seen histologically on an H&E slide. In observations, Omer and Ng kee kwong have shown that there are TLS which do not show expression of CD21 and CD23 expressing cells, which are used as markers of TLS. Therefore, TLS, demonstrated with specific immunostain, represents an underestimate of the total number of TLS. In the study by Silina *et al* [11], the total TLS number can only be identified on histological interpretation on the H&E slides. Although histopathologists have recognized the presence of TLS as Crohn’s-like lymphoid aggregates adjacent to colorectal adenocarcinomas since at least 1990 [10], current methods of detecting and quantifying TLS vary in the literature. Other factors, such as the TLS diameter has been shown to affect the prognosis of colorectal cancer [58]. Therefore, the counting of TLS over a large histological area should be facilitated such that other factors could be integrated in the prognostic determination of the TLS.

We first develop a machine learning automated approach to mimic the identification of the TLS by histopathologists. This had a 92.87% specificity at 95% sensitivity, 88.79% specificity at 98% sensitivity and 84.32% specificity at 99% sensitivity level, implying that the automated approach was able to reproduce histopathologists assessment with great accuracy. This was compared to existing methods of vision recognition and our methodology was superior to those. After the lymphocytes have been segmented, we then used this approach to try to determine the mathematical criteria defining a TLS. Firstly, it appears apparent that there should be a minimum number of lymphocytes within the TLS. In this limited series of TLS, we have shown that the minimum number of lymphocytes is 45. It is also apparent that the TLS has a minimum area and we have shown this to be 6,245*μm*^2^. Visually, we recognise that the lymphocytes are more tightly packed in TLS compared to those in the same tissue, but outside of the TLS. We have here shown that the density of lymphocytes is approximately 3 times those outside of the TLS. The mean density of lymphocytes within a TLS area is 0.0128*μm*^2^ which is much higher compared to 0.004*μm*^2^ in non-TLS regions.

We therefore argue that future studies should use the following criteria for definition of TLS: lymphocyte number within the TLS, the minimum area of the TLS, and density of lymphocytes within a given area. As the density of the lymphocytes within the TLS is much higher than those outside the TLS, the algorithm has demonstrated a distinction between TLS and the tumour infiltrating lymphocytes. The latter are especially relevant to the prognostic factor of breast cancer and therefore, this current trained deep learning model cannot be used to detect regions of tumour infiltrating lymphocytes. A limitation of this current study is that we were not able to correlate TLS density with the patient outcome, owing to the low number of patients in each dataset. Therefore, future studies should correlate the above 3 criteria with outcome of lung cancer patients in large datasets. Based on these findings, it would be possible to adjust the particular values used to define the TLS, to give an optimum definition based on clinical outcome data. Standardisation of TLS density assessment by machine learning will allow the comparison of the host immune response between different subtypes of the same tumour, and between the same tumour across different studies. It will also allow for better correlation between TLS density and outcome.

Another limitation is that this study is based on lung cancer histological images. Future studies are needed to prove that this methodology will be validated in other cancer types and in material from other centers. However, in our experience, the TLS are histologically identical between the different cancer types and we expect this algorithm to be validated in other cancer subtypes too. In our histological experience, lung biopsies are quite narrow and therefore only show part of the TLS, if they were present. Therefore, the smooth outline of the TLS would not be seen in the biopsy and therefore, the algorithm is unlikely to be effective. The model would need to be adapted to detect TLS in biopsies. This can be the subject of another study.

This algorithm provides for the detection of all TLS, but does not make a distinction between the different types of TLS previously described by Silina *et al*. [9–11]. However, we have made observations whereby TLS with germinal centres (secondary follicle-like TLS) constitute 53% of all TLS in a series of lung cancer cases (Omer and Ng kee kwong). We believe that the number of these TLS subtypes would be dependent on the total number of TLS. In this study, we used morphology to detect TLS. A future step would be to use machine learning to quantify TLS of different subtypes.

Our data shows that the performance of the proposed methodology for TLS identification remains high even when the methodology is trained with a limited number of data. The estimated accuracy rates mean that there is a possibility that this AI method can be used in larger scale studies and possibly future clinical diagnostic studies. Although the tumour inflammatory response is assessed on H&E slides using tumour infiltrating lymphocytes, the assessment of TLS is not used in diagnostic practice as there is no precise or standard definition [1]. We therefore believe that our methodology can be used in future studies, including those of non-pulmonary tumour sites. Furthermore, our study provides the foundations for the standardisation of the definition and quantification of the TLS on standard H&E histological images. In addition, we have shown that machine learning can provide a fast and reliable method of quantification. This would possibly lead to its widespread adoption in routine histopathological practice.
